# Evaluating the potential mediating role of ADAMTS13 activity in the relationship between obesity and the severity of COVID-19: A retrospective cohort study

**DOI:** 10.1097/MD.0000000000037806

**Published:** 2024-04-12

**Authors:** Wael Hafez, Asrar Rashid, Hesham Mohamed Abuelsaoud, Mohan Jose, Samy Kishk, Muneir Gador, Tesfalidet Emoshe, Fatema Abdulaal, Nivedita Nair, Muhammad Ahmad, Vanya Jalal Rashid, Youmna Faheem, Steffi John, Sabah Ahmed, Ahmed Daraghmi, Rami Soliman, Ahmed Abdelrahman, Ahmed Ali Mohamed, Mirvat Ghanem

**Affiliations:** a NMC Royal Hospital, Khalifa City, Abu Dhabi, United Arab Emirates; b Internal Medicine Department, Medical Research and Clinical Studies Institute; The National Research Centre, Cairo, Egypt; c National Institute of Chest and Allergy, Egypt; d Internal Medicine Department, Zagazig Faculty of Medicine, Zagazig, Egypt; e Intensive Care Department, Theodor Bilharz Research Institute, AL Warak, Giza Governorate, Egypt.

**Keywords:** ADAMTS-13 activity, COVID-19, fatty liver, liver functions, obesity, viral clearance

## Abstract

Obesity and low enzyme A disintegrin and metalloproteinase with thrombospondin type-1 motif-13 (ADAMTS13) activity have been linked to poor coronavirus disease 2019 (COVID-19). Given that obesity may influence ADAMTS13 activity, it is feasible; however, it remains unclear whether ADAMTS13 activity acts as a mediator between obesity and COVID-19 outcomes. We investigated the link between body mass index (BMI) and COVID-19 outcomes, using ADAMTS13 activity as a mediator. ADAMTS13 activity was measured in 86 hospitalized COVID-19 patients. BMI, ADAMTS13 activity, and COVID-19 outcomes were assessed. Obese patients had a high odds ratio for low ADAMTS13 levels. When different levels of ADAMTS13 activity were considered, the severity of COVID-19 in obese patients was 4.5 times that in the normal BMI group. Furthermore, increased coagulopathy indicators correlated with low ADAMTS13 activity. Patients with elevated ALT and AST levels showed a 3 to 4-fold increase in the chances of low ADAMTS13 activity (OR:3.19, 95% CI:1.22–8.90, *P* = .021; OR:2.17, 95% CI:0.91–5.27, *P* = .082, respectively). When ADAMTS13 activity was considered, obese patients had greater COVID-19 severity and slower viral clearance than those with normal BMI. Low ADAMTS13 activity and impaired liver function are associated with poor COVID-19 outcomes. These findings encourage researchers to use molecular component identification to study the effects of obesity on the von Willebrand factor (VWF)/ADAMTS13 axis, COVID-19 pathogenesis, and outcomes.

## 1. Introduction

The high prevalence of coagulopathy in patients infected with the coronavirus 2019 (COVID-19) pandemic and its well-documented association with the substantial elevation in mortality rates and unmet clinical outcomes have prompted many researchers to investigate coagulopathy-relevant abnormalities, such as elevated D-dimer levels, fibrin degradation product, and prolonged prothrombin time in this patient population.^[1–4]^ Complete autopsies of deceased COVID-19 patients, including postmortem computed tomography, supported by a plethora of studies in critically ill patients, have identified deep venous thrombosis and subsequent pulmonary embolism as hallmarks of pandemic-related deaths.^[5–7]^ Furthermore, patients with COVID-19 frequently have pulmonary and extrapulmonary microthrombotic and thromboembolic indicators as well as significant pulmonary angiogenesis.^[8,9]^

Endothelial cells and megakaryocytes produce Von Willebrand factor (VWF), which is a multimeric plasma glycoprotein.,^[10]^ which is required for both primary hemostasis via platelet and subendothelial collagen adhesion and the intrinsic coagulation cascade via factor VIII (FVIII) stabilization.^[11,12]^ VWF shields FVIII from degradation by activated protein C and localizes FVIII to the plate-let plug and clot formation sites by producing a non-covalently bound VWF-FVIII complex.^[13]^ Because ultralarge VWF (ULVWF) multimers bind to the platelet receptor GPIb-IX-V complex with hyperactivity, they can cause spontaneous platelet aggregation and thrombosis in small arterioles and capillaries.,^[14]^ which is why they should be cleared from plasma as quickly as possible. Plasma VWF levels are governed by a specific proteolytic process mediated by enzyme A disintegrin and metalloproteinase with thrombospondin type-1 motif-13 (ADAMTS13), which cleaves VWF into 2 smaller inactive subunits.^[15]^

Thrombotic thrombocytopenic purpura is a rare life-threatening thrombotic microangiopathy caused by a deficiency in ADAMTS13 enzymatic activity as a consequence of congenital, autosomal recessive, or aberrant autoimmune activity.^[16]^ Owing to the poor enzymatic functionality of ADAMTS13, VWF multimers accumulate, resulting in a dramatic increase in thrombus formation and platelet consumption.^[17]^

Many studies have linked COVID-19 case severity and mortality to hypercoagulability, thought to be caused by a quantitative imbalance between VWF and ADAMTS13 activity.^[17,18]^ The markedly elevated VWF antigen and propeptide ratios suggest reduced VWF clearance.^[19]^ Rodríguez et al revealed an association between elevated VWF and decreased ADAMTS13 levels, and an increased risk of high in-hospital mortality due to COVID-19, implying that ADAMTS13 could be a prognostic marker for COVID-19.^[20]^ In a previous study by our group, we found that reduced ADAMTS13 activity was associated with the development of pneumonia, the need for mechanical ventilation, and the severity of COVID-19; however, there was no statistically significant association with mortality.^[21]^

Although obesity has been recognized as a significant predictor of poor COVID-19 clinical outcomes, especially among the elderly,^[22–24]^ the exact mechanism remains poorly understood. Visceral obesity has been linked to a low-grade chronic inflammatory state and an increased risk of thrombotic manifestations, most likely because adipose tissue is involved in the release of multiple inflammatory agents, including plasminogen activation inhibitor-1, interleukin-6, tumor necrosis factor-, and thrombospondin-1, which affect ULVWF release from endothelial tissue and inhibit its cleavage by ADAMTS13.^[25]^ Increased obesity predisposes individuals to fat accumulation in the liver, leading to nonalcoholic fatty liver disease (NAFLD) and its more severe form, nonalcoholic steatohepatitis, which may progress to liver cirrhosis and hepatocellular carcinoma.^[26]^ Furthermore, several studies have detected ADAMTS13 autoantibodies in obese individuals and patients with chronic liver impairment.^[27–29]^ We hypothesized that obesity-related poor outcomes could be partially related to the effect of obesity on ADAMTS13 activity and aimed in the current study to examine the association between body mass index (BMI) and COVID-19 outcomes while considering ADAMTS13 activity as a potential mediating factor in this loop.

## 2. Materials and methods

### 2.1. Study design and data collection

This was a retrospective analysis of COVID-19 patients over the age of 18 years conducted in accordance with the Helsinki Declaration. After obtaining approval from the ethical institutional review board, the patients’ medical records were reviewed. To protect patient confidentiality, patient identities were deleted during the data processing. The patients’ electronic medical records (INSTA system) were used to collect and analyze demographic data (age, sex, BMI, and underlying comorbid conditions), as well as clinical and biochemical data (clinical symptoms, relevant imaging, laboratory findings, therapeutic interventions, and disease outcomes). Abdominal ultrasonography and baseline laboratory tests performed at the time of admission and throughout admission included inflammatory markers indicative of severe disease (platelet and lymphocyte counts, C-reactive protein [CRP], lactate dehydrogenase [LDH], fibrinogen, D-dimer, complete blood count, liver and kidney function tests, lymphocytic count, and serum ferritin).

### 2.2. Definitions

BMI was divided into 4 categories: underweight (BMI 18.5), normal (BMI 18.5–25), overweight (BMI 25.0 · 30), and obese (BMI 30.0 or higher).^[30]^ The ADAMTS13 activity cutoff value was 70%; findings over this value were regarded as normal, while values below 70% were considered low.^[31]^ Patients admitted to the intensive care unit or cardiac care unit require advanced respiratory support and support of 2 or more organ systems.^[32]^ Time to viral clearance was defined as the time interval between the first positive and first negative polymerase chain reaction (PCR) test from 2 consecutive negative tests. Fatty liver disease was defined as the ultrasound findings of fatty liver disease, whether present or absent. Liver function was defined by abnormal alanine transaminase (ALT) and/or aspartate aminotransferase (AST) levels.

### 2.3. COVID-19 testing

Nasopharyngeal swab RNA was isolated using a Xybio Extraction Kit (Korea). The Bio-Rad Cycler PCR machine from the USA was employed for real-time reverse transcription polymerase chain reaction (RT-PCR), utilizing Solgent 2019-nCoV Real-Time Reverse Transcription PCR Kit, according to the manufacturer instructions.

Viral detection was performed using a CFX-96 plate reader (Bio-Rad). SARS-CoV-2 was detected using RT-PCR. A cycle threshold value (Ct) of <40 was interpreted as a negative result, whereas a Ct value > 40 was considered positive.

### 2.4. Plasma activity of ADAMTS13

The examination was carried out at the United Arab Emirates National Reference Laboratory using order code 117913. ADAMT13 activity in plasma was quantified using liquid chromatography-tandem mass spectrometry analysis. ADAMTS13 activity was ascertained based on its capacity to cleave a synthetic polypeptide substrate (VWF73) introduced into plasma specimens. The threshold for ADAMT13 activity was set at 66.8%. Findings surpassing this value were categorized as normal, whereas results below 66.8% indicated low ADAMTS13 activity. The assessment was designed by LabCorp Burlington, located in 1447 York City, Burlington, NC, USA.

### 2.5. Statistical analysis

Data were analyzed statistically using R Software (version 4.2.1; 23–06-2022) - “Funny-Looking Kid.” Means, standard deviations, and ranges were used for normally distributed data. The median and interquartile range were used for skewed data. Frequencies (n) and percentages (%) are used to represent categorical data.

A univariate logistic regression model was used to investigate the relationship between all the factors and ADAMTS13 levels. When different ADAMTS13 levels were investigated, variables with p-values in the univariate model were included in a multivariate logistic regression to investigate the collective connection between BMI and COVID-19 severity and mortality. The Kaplan–Meier curve and log-rank test were used to examine the relationship between BMI and time to viral clearance while considering varying levels of ADAMTS13 activity. Uncorrected and adjusted hazard ratios were calculated using Cox regression analysis. The confidence interval was set at 95%, and a *P* value was considered significant when it was <.05.

## 3. Results

### 3.1. Patient demographics

The medical records of 86 COVID-19 patients who were tested for plasma ADAMTS13 activity were reviewed. Males accounted for 85.1% of the population, while females accounted for 14.9%. The mean ± standard deviation age of the study population was 43.1 ± 10.1, while it was 53 ± 8.5 for deceased patients compared to 42.3 ± 9.8 for recovered patients (*P* = .013). The median (inter-quartile range) length of hospital stay was 10 days (range: 7–14 days). Although 13% of patients were admitted to critical care units, all deceased patients died in the intensive care unit (*P* < .001). Approximately 35% of the study population had at least one comorbidity, with diabetes and hypertension being the most common. Meanwhile, 70% of the study population had a BMI ≥ 25. Most patients (62%) presented with mild to moderate COVID-19 severity. As shown in Table 1

**Table 1:** Demographic characteristics of recovered and deceased participants.

Demographic characters of the study participants	Overall, N = 86†	Recovered, N = 80†	Died, N = 6†	*P* value*
Age	44 (36, 50)	42 (36, 48)	52 (47, 61)	**.013**
≤44	45 (52%)	45 (56%)	0 (0%)	**.010**
> 44	41 (48%)	35 (44%)	6 (100%)	
SEX				.195
Female	12 (14%)	10 (13%)	2 (33%)	
Male	74 (86%)	70 (88%)	4 (67%)	
RACE				.277
Asian	11 (13%)	11 (14%)	0 (0%)	
Black	71 (83%)	66 (83%)	5 (83%)	
White	4 (4.7%)	3 (3.8%)	1 (17%)	
Length of hospital stay	10 (7, 14)	10 (7, 14)	0 (0, 0)	>.999
≤10 d	43 (56%)	43 (56%)	0 (0%)	>.999
> 10 d	34 (44%)	34 (44%)	0 (0%)	
Comorbidities	31 (36%)	28 (35%)	3 (50%)	.663
HTN	17 (20%)	15 (19%)	2 (33%)	.339
DM	20 (23%)	18 (23%)	2 (33%)	.620
CVS	5 (5.8%)	4 (5.0%)	1 (17%)	.310
BMI	27.6 (24.8, 31.2)	27.6 (24.7, 31.1)	28.7 (26.3, 31.4)	.832
Normal	24 (28%)	23 (29%)	1 (17%)	.682
Obese	27 (31%)	24 (30%)	3 (50%)	
Overweight	34 (40%)	32 (40%)	2 (33%)	
Underweight	1 (1.2%)	1 (1.3%)	0 (0%)	
Severity index				**.003**
Non severe	52 (60%)	52 (65%)	0 (0%)	
Severe	34 (40%)	28 (35%)	6 (100%)	
ICU admission				**<.001**
No	75 (87%)	75 (94%)	0 (0%)	
Yes	11 (13%)	5 (6.3%)	6 (100%)	

*P* - value <0.05.

BMI = body mass index, CVS = cardiovascular disease, DM = diabetes mellitus, HTN = hypertension, ICU = intensive care unit, IQR = interquartile range, N = sample size.

† Median (IQR); n (%).

* Wilcoxon rank sum test; Fisher’s exact test.

### 3.2. Laboratory findings

While 52% of the study population had low ADAMTS13 levels, 83% of deceased patients had the same low levels. Deceased patients showed significantly abnormal levels of neutrophils, lymphocytes, hemoglobin, CRP, D-dimer, and liver function test (LFT) results. The coagulation profile, presented as fibrinogen and ferritin levels, was significantly elevated in the deceased group. As shown in Table 2.

**Table 2:** Laboratory results distributed between recovered and deceased participants.

Laboratory results	Overall, N = 86†	Recovered, N = 80†	Died, N = 6†	*P* value*
ADAMTS-13 activity	69 (55, 83)	71 (56, 84)	56 (48, 60)	.107
Low	44 (51%)	39 (49%)	5 (83%)	.203
Normal	42 (49%)	45 (51%)	1 (17%)	
CRP	23 (4, 95)	21 (4, 90)	99 (79, 108)	**.036**
High (>10)	58 (68%)	52 (66%)	6 (100%)	.170
Normal (1–10)	27 (32%)	27 (34%)	0 (0%)	
D_DIMER	0.5 (0.3, 1.1)	0.4 (0.3, 0.9)	13.6 (10.5, 18.1)	**<.001**
High (>0.5)	41 (48%)	35 (44%)	6 (100%)	**.010**
Normal (≤0.5)	44 (52%)	44 (56%)	0 (0%)	
LDH	270 (211, 426)	254 (206, 397)	646 (529, 1041)	**<.001**
High (>280)	41 (48%)	35 (44%)	6 (100%)	**.010**
Normal (140–280)	45 (52%)	45 (56%)	0 (0%)	
ALT	44 (30, 64)	44 (30, 64)	44 (30, 59)	.887
High (>55)	26 (31%)	24 (30%)	2 (40%)	.643
Normal (7–55)	58 (69%)	55 (70%)	3 (60%)	
AST	43 (27, 56)	40 (27, 55)	47 (46, 62)	.108
High (>48)	44 (52%)	39 (49%)	5 (100%)	.056
Normal (8–48)	40 (48%)	40 (51%)	0 (0%)	
FIBRINOGEN	542 (365, 666)	527 (360, 650)	784 (631, 907)	**.009**
High (>400)	61 (71%)	55 (69%)	6 (100%)	.175
Normal (200–400)	25 (29%)	25 (31%)	0 (0%)	
FERRITIN	351 (149, 1033)	308 (137, 828)	1723 (1654, 1910)	**<.001**
High (>336)	44 (52%)	38 (48%)	6 (100%)	**.026**
Normal (24–336)	41 (48%)	41 (52%)	0 (0%)	
White blood cells	6.00 (4.73, 7.77)	6.11 (4.82, 7.82)	5.16 (4.53, 5.85)	.170
Hemoglobin	13.90 (12.80, 15.08)	14.20 (13.00, 15.13)	12.50 (11.38, 13.03)	**.019**
Platelets	285 (233, 417)	287 (232, 428)	268 (248, 289)	.297
Interleukin-6	48 (21, 163)	40 (20, 99)	1200 (712, 1688)	.068
Creatinine	0.86 (0.75, 1.01)	0.86 (0.73, 1.00)	1.02 (0.88, 1.24)	.082
Neutrophil count	61 (53, 75)	61 (52, 73)	83 (78, 86)	**<.001**
Lymphocyte count	27 (17, 36)	28 (19, 37)	12 (10, 16)	**.001**
NLR	2.35 (1.43, 4.44)	2.14 (1.38, 3.87)	6.70 (4.98, 9.10)	**.001**
Prothrombin time	13.90 (13.00, 15.00)	14.00 (13.00, 15.00)	13.40 (13.20, 13.70)	.709
INR	1.00 (0.94, 1.09)	0.99 (0.94, 1.08)	1.08 (1.01, 1.13)	.139
Time to viral clearance	17 (12, 25)	17 (13, 26)	19 (14, 22)	.884

*P* - value <0.05.

ADAMTS13 = a disintegrin and metalloproteinase with a thrombospondin type 1 motif, member 13, ALT = alanine transaminase, AST = aspartate aminotransferase, CRP = C-reactive protein, INR = international normalized ratio, IQR = interquartile range, LDH = lactate dehydrogenase, N = sample size, NLR = neutrophil-lymphocyte ratio.

† Median (IQR); n (%).

* Wilcoxon rank sum test; Fisher’s exact test; Wilcoxon rank sum exact test.

### 3.3. Logistic regression analysis

#### 3.3.1. ADAMTS13 activity and LFTs

Compared to patients with normal levels, patients with deranged liver function (elevated ALT and AST levels) showed a 3- to 4-fold increase in the likelihood of low ADAMTS13 activity (OR, 3.19; 95% CI:1.22–8.90, *P* = .021; OR:2.17, 95% CI:0.91–5.27, *P* = .082, respectively). In univariate analysis, patients with elevated LDH levels showed a non-significant trend of low ADAMTS13 activity compared to those with normal levels, whereas multivariate analysis indicated high significance.

A statistically significant likelihood of low ADAMTS13 activity was associated with elevated levels of the inflammatory and coagulopathy markers CRP (OR:3.04, 95% CI:1.19–8.22, *P* = .023), D-dimer (OR:3.04, 95% CI:1.19–8.22, *P* = .023), fibrinogen (OR:2.39, 95% CI:0.93–6.47, *P* = .075), and ferritin (OR:3.35, 95% CI:1.40–8.35, *P* = .008) (Table 3).

**Table 3:** Logistic regression to show the predictors of ADAMTS levels.

Dependent: ADAMTS-13 level		Crude OR (95% CI, *P* value)	Adjusted OR (95% CI, *P* value)
Age	≤ 44	-	-
	> 44	1.21 (0.52–2.84, *P* = .659)	2.16 (0.25–23.92, *P* = .491)
Sex	Female	-	-
	Male	1.56 (0.46–5.69, *P* = .480)	7837.58 (54.8-inf, *P* = .003)
BMI	Normal	-	-
	Obese	2.08 (0.69–6.58, *P* = .200)	5.04 (0.35–95.59, *P* = .243)
	Overweight	2.11 (0.74–6.32, *P* = .170)	6.53 (0.44–174.68, *P* = .197)
	Underweight	inf (0.00-inf, *P* = .991)	inf (0.00-inf, *P* = .988)
Length of hospital stay	≤ 10 d	-	-
	> 10 d	0.49 (0.19–1.21, *P* = .127)	0.03 (0.00–0.32, *P* = .012)
Comorbidities	No	-	-
	Yes	1.03 (0.43–2.50, *P* = .950)	0.01 (0.00–1.59, *P* = .111)
Hypertension	No	-	-
	Yes	1.47 (0.51–4.47, *P* = .482)	3.64 (0.11–209.84, *P* = .484)
Diabetes mellitus	No	-	-
	Yes	1.22 (0.45–3.41, *P* = .695)	41.07 (0.76–10260.38, *P* = .114)
Cardiovascular diseases	No	-	-
	Yes	1.46 (0.23–11.55, *P* = .685)	1532.88 (0.55-inf, *P* = .315)
Platelet count	Normal (150–450)	-	-
	Thrombocytopenia (<150)	2.33 (0.21–51.68, *P* = .498)	1.39 (0.02–198.50, *P* = .882)
	Thrombocytosis (>450)	2.33 (0.80–7.41, *P* = .129)	7.70 (0.50–203.52, *P* = .167)
C-reactive protein	Normal (1–10)	-	-
	High (>10)	3.04 (1.19–8.22, *P* = .023)	24.71 (1.66–886.60, *P* = .037)
D-dimer	Normal (≤0.5)	-	-
	High (>0.5)	3.06 (1.28–7.60, *P* = .013)	2.31 (0.22–29.84, *P* = .489)
ALT level	Normal	-	-
	Elevated	3.19 (1.22–8.90, *P* = .021)	1.48 (0.09–32.78, *P* = .780)
AST level	Normal	-	-
	Elevated	2.17 (0.91–5.27, *P* = .082)	1.61 (0.13–23.92, *P* = .705)
Ferritin	Normal (24–336)	-	-
	High (>336)	3.35 (1.40–8.35, *P* = .008)	0.01 (0.00–0.50, *P* = .042)
Fibrinogen	Normal (200–400)	-	-
	High (>400)	2.39 (0.93–6.47, *P* = .075)	0.99 (0.04–18.76, *P* = .993)
Severity index	Nonsevere	-	-
	Severe	4.82 (1.92–12.98, *P* = .001)	277.88 (5.30–61821.13, *P* = .015)
Time to viral clearance		1.02 (0.98–1.07, *P* = .285)	0.92 (0.80–1.02, *P* = .151)
Creatinine		1.22 (0.23–6.57, *P* = .811)	0.22 (0.00–17.58, *P* = .514)
Neutrophil count		1.02 (0.99–1.05, *P* = .301)	2.19 (1.35–4.41, *P* = .007)
Lymphocyte count		0.99 (0.96–1.03, *P* = .746)	3.02 (1.61–7.67, *P* = .004)

ADAMTS13 = disintegrin and metalloproteinase with a thrombospondin type 1 motif, member 13, BMI = body mass index, CI = confidence interval.

#### 3.3.2. ADAMTS13 activity, BMI, and mortality

Although a high odds ratio of low ADAMTS13 levels was observed among obese and overweight patients, ADAMTS13 activity did not demonstrate a statistically significant relationship with other BMI categories (Table 3). After adjusting for BMI, non-significantly higher odds of mortality were observed in patients with low ADAMTS13 activity than in those with normal ADAMTS13 activity levels (Table 4).

**Table 4:** Logistic regression to show the relationship between ADMTS level, BMI status, and death due to COVID-19.

Dependent: Death		Crude OR (95% CI, *P* value)	Adjusted OR (95% CI, *P* value)
ADAMTS-13 level	Normal	-	-
	Low	5.26 (0.80–103.12, *P* = .138)	5.14 (0.76–101.96, *P* = .147)
BMI	Normal	-	-
	Obese	2.88 (0.34–60.46, *P* = .375)	2.27 (0.25–48.93, *P* = .500)
	Overweight	1.44 (0.13–32.03, *P* = .772)	1.10 (0.10–25.22, *P* = .938)
	Underweight	inf (0- inf, *P* = .996)	inf (0- inf, *P* = .995)

ADAMTS13 = disintegrin and metalloproteinase with a thrombospondin type 1 motif, member 13, BMI = body mass index, CI = confidence interval.

#### 3.3.3. ADAMTS13 activity, BMI, and case severity

The likelihood of low ADAMTS13 activity was statistically significant among severe cases (*P* = .001). The severity of COVID-19 in obese patients was 4.5-fold that in the comparable group when considering different levels of ADAMTS13 activity (*P* = .001) (Table 5).

**Table 5:** Logistic regression to show the relationship between ADMTS level, BMI status, and the severity index of COVID-19 infection.

Dependent: severity index		Crude OR (95% CI, *P* value)	Adjusted OR (95% CI, *P* value)
ADAMTS-13 levels	Normal	-	-
	Low	4.82 (1.92–12.98, *P* = .001)	5.58 (2.06–16.72, *P* = .001)
BMI	Normal	-	-
	Obese	5.10 (1.59–18.25, *P* = .008)	4.84 (1.36–19.27, *P* = .018)
	Overweight	1.43 (0.45–4.86, *P* = .545)	1.08 (0.30–3.95, *P* = .908)
	Underweight	0.00 (NA-inf, *P* = .992)	0.00 (NA-inf, *P* = .992)

ADAMTS13 = disintegrin and metalloproteinase with a thrombospondin type 1 motif, member 13, BMI = body mass index, CI = confidence interval.

### 3.4. The association between the rate of viral clearance and BMI while considering ADAMTS13 activity

The rate of viral clearance significantly decreased in obese patients by 50% compared to those with normal BMI after accounting for different plasma ADAMTS13 activity levels (hazard ratios:0.71, 95% CI:0.45–1.12, *P* = .138). In addition, patients with low ADAMTS13 activity showed a non-significant prolonged viral clearance rate compared to those with normal ADAMTS13 activity after accounting for different BMI categories (Table 6).

**Table 6:** Cox proportional hazard regression to show the predictors of viral clearance probability.

Dependent:		Crude HR (95% CI, *P* value)	Adjusted HR (95% CI, *P* value)
ADAMTS-13 level	Normal	-	-
	Low	0.71 (0.45–1.12, *P* = .138)	0.76 (0.48–1.19, *P* = .228)
BMI	Normal	-	-
	Obese	0.59 (0.33–1.07, *P* = .082)	0.60 (0.34–1.09, *P* = .094)
	Overweight	0.87 (0.50–1.51, *P* = .628)	0.89 (0.51–1.54, *P* = .671)
	Underweight	0.22 (0.03–1.68, *P* = .144)	0.25 (0.03–1.93, *P* = .184)

Note: Only one participant was underweight; therefore, the program failed to detect the exact odds ratio and 95% confidence interval and gave infinity.

ADAMTS13 = disintegrin and metalloproteinase with a thrombospondin type 1 motif, member 13, BMI = body mass index, CI = confidence interval.

**Figure 1.**
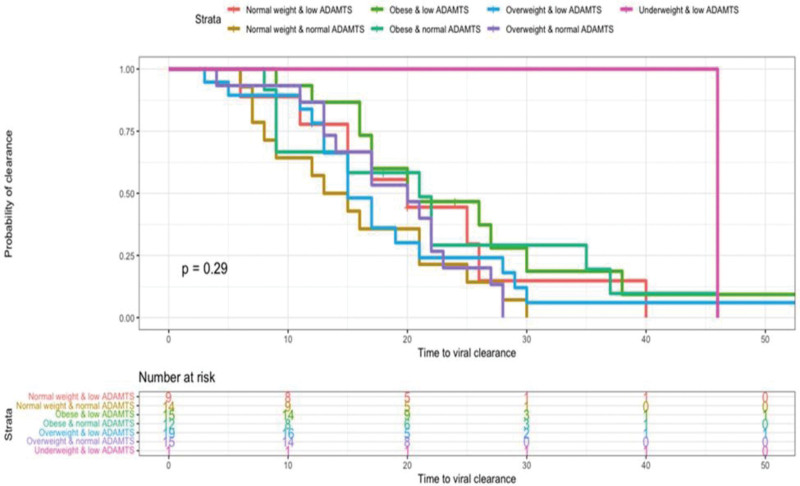
Kaplan–Meier curve showing the time to viral clearance distributed across BMI groups by considering ADAMTS status. ADAMTS = the enzyme A disintegrin and metalloproteinase with thrombospondin, BMI = body mass index.

The median time to viral clearance in patients with normal BMI/normal ADAMTS13 was 16 days compared to 19 days in patients with normal BMI/low ADAMTS13, while for obese/normal ADAMTS13, it was 22 days compared to 26 days in obese patients with low ADAMTS13. The Kaplan–Meier survival curve revealed a non-significant difference between different BMI categories when considering different ADAMTS13 activity levels regarding the time to viral clearance (*P* = .29) (Fig. 1).

## 4. Discussion

In this retrospective study, we investigated the correlation between BMI and the outcomes of COVID-19 while considering ADAMTS13 activity in 87 hospitalized adult COVID-19 patients. Despite the observed association between low ADAMTS13 activity and an increased risk of COVID-19 severity, we did not find a significant correlation between patient BMI and ADAMTS13 activity.

We found significantly high odds of low ADAMTS13 activity in patients with elevated LDH, AST, and ALT levels. We also statistically correlated high BMI, low ADAMTS13 activity, and poor COVID-19 outcome. Numerous studies have documented the negative impact of obesity on COVID-19 treatment outcomes and the need for critical care admission and mechanical ventilation among these patients^[33,34]^; however, a clear explanation has yet to be identified. Lighter et al found that COVID-19 patients aged < 60 years with a BMI ≥ 30 kg/m^2^ were more likely (1.8–3.6-fold, *P* = .0001) to be admitted to acute and critical care than same-aged patients with a BMI < 30.^[35]^ The mounting mortality rates and need for intensive care among obese patients of various grades prompted the American Heart Association to advise rigorous adherence to precautionary measures in this special population during the COVID-19 pandemic.^[36]^ In our study, we observed a significant correlation [OR:4.5, *P* = .015] between obesity (30–35 kg/m^2^) and COVID-19 severity, with no correlation with other obesity grades, probably because of the low number of patients in these groups.

Low-grade chronic inflammation caused by obesity is a risk factor for various cardiovascular diseases. Obesity has also been linked to acquired thrombotic thrombocytopenic purpura induced by ADAMTS13 deficiency.^[27,37]^ While we did not find a significant correlation between BMI and ADAMTS13 activity in our study, low ADAMTS13 activity was found to be a significant predictor of prolonged time to viral clearance, after adjusting for BMI. Obese patients with low ADAMTS13 activity had a significantly longer time to viral clearance. Our finding regarding the relationship between BMI and ADAMTS13 was contradictory to the results of the African-PREDICT study, which reported a negative association between BMI and ADAMTS13 levels, likely because of the small number of patients among different BMI categories in our study group.^[38]^ Meanwhile, Thangaraju et al replicated our findings regarding the relationship between poor COVID-19 outcomes and low ADAMTS13 activity and reported low ADAMTS13 activity in critically ill and deceased COVID-19 patients and replicated our findings of an inverse correlation between ADAMTS13 levels and D-dimer, fibrinogen, and ferritin.^[37]^

The time to viral clearance was significantly prolonged in obese patients with low ADAMTS13 activity; the same result was reported by our research team in a previous study.^[21]^ Delayed viral clearance and poor outcomes in obese patients are hallmark findings of several studies.^[39,40]^ Reduced ADAMTS13 activity was one of the most obvious drivers of unmet treatment outcomes in this special patient population, highlighting the importance of rigorous assessment of hospitalized obese COVID-19 patients, regardless of disease severity.

In the same context, and from a different perspective, we found significantly lower ADAMTS13 activity in cases of elevated ALT and AST levels, which could be explained by viral hepatitis or the most common NAFLD associated with obesity, reflecting the link between obesity and low ADAMTS13 activity and attributed to cytokinemia, the presence of plasma ADAMTS13 inhibitors, or reduced and defective ADAMTS13 synthesis in the impaired liver.^[41,42]^ Although we cannot claim that abnormal liver function in obese COVID-19 patients is solely due to steatohepatitis, as it could be due to other factors such as direct viral effects, medication effects, and/or toxicity from cytokinemia, the finding that only abnormal liver function was significantly associated with higher odds of poor outcome suggests that NAFLD without steatohepatitis (normal LFTs) is unlikely to affect the outcome of patients. However, NAFLD combined with steatohepatitis may influence COVID-19 outcomes.

Although our study was one of the few to prove the intimate link between low ADAMTS13 activity in obese patients or patients with fatty liver and unmet treatment outcomes in COVID-19, it is still limited by the retrospective nature of the study, the relatively small number of patients with extreme obesity grades, and fluctuations in ADAMTS13 activity during the hospitalization course. Future larger, well-designed research to explore the same is highly recommended.

## 5. Conclusion

We conclude that low ADAMTS13 activity in obese individuals is correlated with a high risk of COVID-19 severity and slower viral clearance than in those with normal BMI, but without a significant correlation between patient BMI and ADAMTS13 activity.

These findings suggest that obesity-related medical conditions, such as nonalcoholic liver disease with steatohepatitis, can impair ADAMTS 13 activity. Low ADAMTS13 activity and impaired liver function were associated with disease severity, poor therapeutic response, clinical recovery delays, viral clearance delays, and increased mortality rates.

Additionally, these findings have motivated the adoption of specific treatment approaches that rely on the rapid and accurate identification of molecular components that may have a detrimental impact on treatment outcomes. Further research on the VWF and ADAMTS13 axes in larger cohorts is needed to better understand how obesity interacts with COVID-19 pathogenesis and outcomes.

## Acknowledgments

We would like to express our sincere gratitude to David Hardley, Alan Stewart, Prakash Janardan, Maki Hamad, Mouhamad Al Zouhby, and the NMC Clinical Research team, Gayathri Rahul, Rohit Dusane, Shailesh Perdakar, and Monica Jadhav for their help, guidance, and support. We would like to express our gratitude to the Global Medical Agency for Research and Statistics (Global-MARS) for their great statistical and editorial support.

## Authors contributions

**Conceptualization:** Wael Hafez.

**Data curation:** Wael Hafez, Asrar Rashid, Hesham Mohamed Abuelsaoud, Mohan Jose, Samy Kishk, Muneir Gador, Tesfalidet Emoshe, Fatema Abdulaal, Nivedita Nair, Muhammad Ahmad, Vanya Jalal Rashid, Youmna Faheem, Steffi John, Sabah Ahmed, Ahmed Daraghmi, Rami Soliman, Ahmed Abdelrahman, Ahmed Ali Mohamed, Mirvat Ghanem.

**Formal analysis:** Wael Hafez, Asrar Rashid, Hesham Mohamed Abuelsaoud, Samy Kishk, Tesfalidet Emoshe, Fatema Abdulaal, Nivedita Nair, Muhammad Ahmad, Youmna Faheem, Steffi John, Ahmed Daraghmi, Rami Soliman, Ahmed Abdelrahman, Ahmed Ali Mohamed, Mirvat Ghanem.

**Investigation:** Wael Hafez, Asrar Rashid, Hesham Mohamed Abuelsaoud, Mohan Jose, Samy Kishk, Muneir Gador, Tesfalidet Emoshe, Fatema Abdulaal, Nivedita Nair, Muhammad Ahmad, Vanya Jalal Rashid, Youmna Faheem, Sabah Ahmed, Ahmed Daraghmi, Rami Soliman, Ahmed Abdelrahman, Ahmed Ali Mohamed.

**Methodology:** Hesham Mohamed Abuelsaoud, Mohan Jose, Muneir Gador, Tesfalidet Emoshe, Nivedita Nair, Muhammad Ahmad, Vanya Jalal Rashid, Steffi John, Sabah Ahmed, Ahmed Daraghmi, Rami Soliman, Mirvat Ghanem.

**Project administration:** Wael Hafez, Ahmed Abdelrahman.

**Resources:** Mohan Jose, Samy Kishk, Youmna Faheem, Sabah Ahmed.

**Software:** Muneir Gador, Fatema Abdulaal.

**Supervision:** Wael Hafez, Asrar Rashid, Samy Kishk, Tesfalidet Emoshe, Vanya Jalal Rashid.

**Validation:** Hesham Mohamed Abuelsaoud, Muhammad Ahmad.

**Writing – original draft:** Wael Hafez.

**Writing – review & editing:** Wael Hafez, Asrar Rashid, Hesham Mohamed Abuelsaoud, Mohan Jose, Samy Kishk, Muneir Gador, Tesfalidet Emoshe, Fatema Abdulaal, Nivedita Nair, Muhammad Ahmad, Vanya Jalal Rashid, Youmna Faheem, Steffi John, Sabah Ahmed, Ahmed Daraghmi, Rami Soliman, Ahmed Abdelrahman, Ahmed Ali Mohamed, Mirvat Ghanem.
